# The *Deinococcus radiodurans* DR1245 Protein, a DdrB Partner Homologous to YbjN Proteins and Reminiscent of Type III Secretion System Chaperones

**DOI:** 10.1371/journal.pone.0056558

**Published:** 2013-02-18

**Authors:** Cédric Norais, Pascale Servant, Claire Bouthier-de-la-Tour, Pierre-Damien Coureux, Solenne Ithurbide, Françoise Vannier, Philippe P. Guerin, Charles L. Dulberger, Kenneth A. Satyshur, James L. Keck, Jean Armengaud, Michael M. Cox, Suzanne Sommer

**Affiliations:** 1 Department of Biochemistry, University of Wisconsin College of Agriculture and Life Sciences, Madison, Wisconsin, United States of America; 2 Ecole polytechnique, Laboratoire de Biochimie, Centre national de la recherche scientifique, Palaiseau, France; 3 Univ. Paris-Sud, Institut de Génétique et Microbiologie (Bât. 409), UMR8621, Orsay, France; 4 Centre national de la recherche scientifique, Orsay, France; 5 Commissariat à l’énergie atomique et aux énergies alternatives, Direction des Sciences du Vivant, Institut de Biologie Environnementale et Biotechnologie, Lab Biochim System Perturb, Bagnols-sur-Cèze, France; 6 Department of Biomolecular Chemistry, University of Wisconsin School of Medicine and Public Health, Madison, Wisconsin, United States of America; University of Saskatchewan, Canada

## Abstract

The bacterium *Deinococcus radiodurans* exhibits an extreme resistance to ionizing radiation. A small subset of *Deinococcus* genus-specific genes were shown to be up-regulated upon exposure to ionizing radiation and to play a role in genome reconstitution. These genes include an SSB-like protein called DdrB. Here, we identified a novel protein encoded by the *dr1245* gene as an interacting partner of DdrB. A strain devoid of the DR1245 protein is impaired in growth, exhibiting a generation time approximately threefold that of the wild type strain while radioresistance is not affected. We determined the three-dimensional structure of DR1245, revealing a relationship with type III secretion system chaperones and YbjN family proteins. Thus, DR1245 may display some chaperone activity towards DdrB and possibly other substrates.

## Introduction

The bacterium *Deinococcus radiodurans* is known for its exceptional resistance to the lethal effects of ionizing radiation (IR), ultraviolet light and many other DNA-damaging agents. *D. radiodurans* is able to withstand 10,000 Gy of γ-radiation, a dose which shatters the genome into hundreds of fragments, without loss of viability. Since its first discovery in the 1950s, different mechanisms have been proposed to explain the extreme radioresistance of this microorganism (for reviews, see [1]–[3]). *Deinococcus* has a fairly standard complement of bacterial DNA repair proteins, leading to speculation that novel DNA repair processes are not sufficient to explain the IR resistance phenotype [4], [5]. Extensive evidence has been put forward that protection against protein oxidation makes the major contribution to this phenotype [4]–[7]. However, upon irradiation, a small but significant suite of proteins is induced in *Deinococcus* [8], [9], and these proteins have a demonstrable effect on radioresistance [9]–[14]. All of these proteins are induced as part of a coordinately regulated response to extreme levels of DNA damage.

DdrB is among the genes that are most highly up-regulated following irradiation [9]. DdrB belongs to a novel class of single-stranded DNA binding protein that comprises a novel fold that is structurally and topologically distinct from all other single-stranded binding (SSB) proteins [15], [16]. This unique ssDNA binding function in response to severe DNA damage, suggests a distinct role for DdrB, which may function in protection of ssDNA, but may also exhibit more specialized roles in protein recruitment. Thus, we initiated an effort to identify proteins that interact with DdrB.

We identified DR1245, a protein of unknown function conserved only in the *Deinococcaceae*, as a DdrB partner in cells recovering from ionizing radiation treatment. Our study indicates that cells devoid of DR1245 grow more slowly under normal (unstressed) growth conditions than wild-type cells, but are as radioresistant as wild type. The DR1245 structure revealed a strong structural homology to an YbjN homolog and Type III secretion system chaperones.

## Results

### DR1245 interacts with DdrB

To identify *in vivo* partners of the DdrB protein, we used a tandem affinity purification (TAP) method in which two tags are fused to the target protein of interest (bait) and the interacting partners (preys) are isolated by using two successive affinity purification steps. The bait proteins were tagged at their C-terminal end with a SPA motif containing the 3xFLAG epitope and the calmodulin binding protein (CBP) separated by a tobacco etch virus (TEV) protease cleavage site [17]. The mutated alleles were inserted at their original genetic locus so that the fusion proteins were expressed under the control of their native promoter. Co-purified proteins were identified by tandem mass spectrometry after resolving the samples on sodium dodecyl sulphate (SDS)-polyacrylamide gels. This procedure was first validated with a pilot experiment in which we purified the *Deinococcal* RNA polymerase holoenzyme using the RpoB protein as bait. In addition to the tagged subunit β (RpoB), the isolated complex contained the other expected subunits, namely α (RpoA), β’ (RpoC), and σ^70^ (RpoD) (Figure S1).

**Figure 1:**
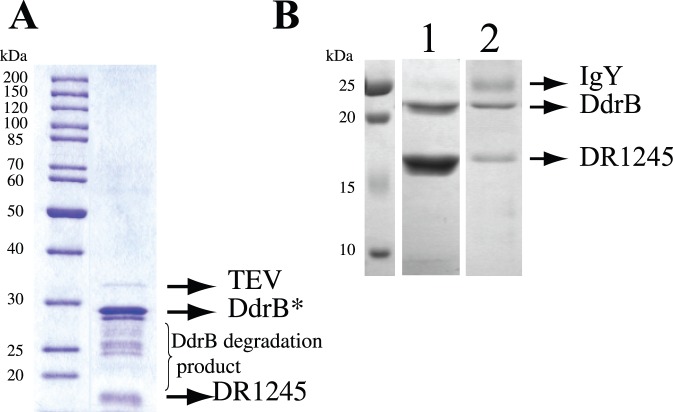
DR1245, an interactant of DdrB. **A.** SDS-PAGE analysis of protein complexes purified from GY12830 strain expressing a tagged DdrB-SPA protein. Purification of protein complexes containing DdrB-SPA was carried out from irradiated cells (3,800 Gy) at 45 minutes after irradiation. * This band corresponds to the tagged DdrB with calmodulin binding protein. **B.** Purified native DdrB (non-tagged) (10 nmol) linked to streptavidin agarose beads using biotinylated anti-DdrB IgY were incubated with 10 nmol purified native (non-tagged) *D. radiodurans* DR1245 protein. Following extensive washes with PBS-Tween, proteins that remained associated to DdrB were eluted using 10x PBS. Proteins contained in the flow-through (1) and elution (2) fractions were analyzed on a 12% SDS-PAGE revealed using Coomassie blue staining. Positions of DdrB, DR1245 and the small subunit of the IgY are indicated.

The DdrB-SPA protein used as bait for TAP-tag analysis is induced about four-fold at early times after gamma irradiation and fully functional [14]. Therefore, we searched for DdrB partners from cells recovering from gamma irradiation (3,800 Gy γ-irradiation dose, 45 min post-irradiation). Figure 1A shows the presence in the sample of the tagged DdrB protein (28 kDa) and its degradation products as certified by tandem mass spectrometry, as well as a protein with a probable molecular weight below 20 kDa. We identified the latter as DR1245 protein in all three independent experiments performed through the detection of 8, 4 and 4 peptide sequences (55, 12, and 7 MS/MS spectra, respectively) (Table S1). DR1245 was not retrieved when RpoB or β-galactosidase were used as bait using the same TAP-tag methodology (data not shown).

To confirm interaction between DdrB and DR1245, we unsuccessfully tried to construct strains containing a functional fusion of DR1245 to either an N-terminal or a C-terminal SPA-tag. Thus, DdrB interaction with DR1245 was further investigated using purified native proteins. The purified native DR1245 protein behaved on a Sephacryl S-300 16/60 HR (Amersham Biosciences) gel filtration column (performed as described in [15]) as a dimer with an apparent size of 43 kDa. Immunopulldown assays provided evidence for a direct interaction between DdrB and DR1245 when anti-DdrB antibodies were used (Figure 1B). However, the interaction with DdrB could not be detected in the reciprocal experiment using anti-DR1245 antibodies in which none of the tested proteins remained associated to DR1245 (results not shown). It is possible that the anti-DR1245 IgY antibody, which is significantly larger than DR1245 (∼180 kDa and ∼43 kDa respectively), sterically prevents DR1245 from interacting with DdrB.

**Figure 2:**
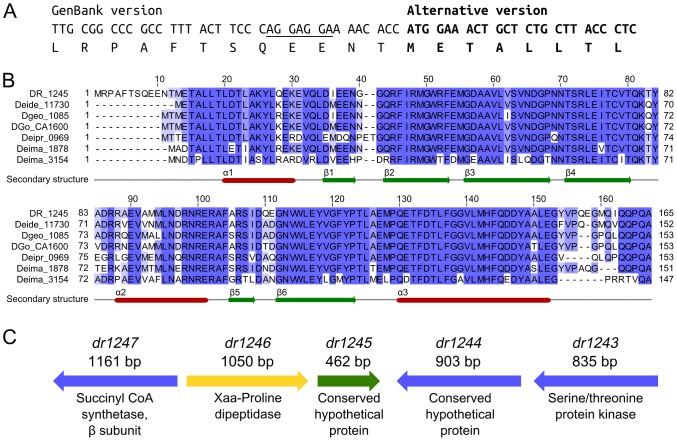
*D. radiodurans* DR1245 protein is shorter than previously annotated. A. Nucleotide sequence of the beginning of *dr1245* showing the ribosome binding site (underlined) upstream of the ATG initiation codon and encoding a DR1245 protein shorter than presently indicated in GenBank (AAF10822.1). **B.** DR1245 homologues among Deinococcacae.DR1245 homologues from sequenced Deinococcaceae (*Deinococcus radiodurans*, DR_1245; *D. deserti*, Deide_11730; *D. geothermalis*, Dgeo_1085; *D. gobiensis*, DGo_CA1600; *D. maricopensis*, Deima_1878; *D. maricopensis*, Deima_3154; *D. proteolyticus*, Deipr_0969) were aligned using MUSCLE [26] with JAL view [27] and represented using the Percentage Identity coloring. Secondary structures determined for DR1245 in this study are indicated below the alignment. **C.** Genome context of the *D. radiodurans dr1245.*

DR1245 was hitherto annotated as a hypothetical protein of 165 amino acids and its theoretical molecular weight was 18903 Da, with an open reading frame beginning at an infrequently used TTG initiation codon [18]. However, *in silico* sequence analyses and mass spectrometry data indicated that this annotation was incorrect. We identified with a confident score the semi-tryptic peptide [METALLTLDTLAK] at *m/z* 718.3895 (dicharged ion, mass error 0.38 ppm) corresponding to the N-terminus of the protein. Figure S2 shows the corresponding assigned MS/MS spectrum with almost complete *b* and *y* ion series. This peptide cannot result from a strict trypsin proteolysis but rather from a translational start at an ATG codon located 12 codons downstream from the previously annotated TTG start (Figure 2A). Furthermore, we identified for the *dr1245* gene a potential ribosomal binding site, AGGAGGA, at an optimal distance of eight nucleotides upstream the mass-spectrometry certified ATG initiation codon (Figure 2A). A BLAST search in the non-redundant protein sequences from NCBI indicates the presence of one DR1245 homologue in most *Deinococcaceae* family members (*D. deserti*, *D. gobiensis*, *D. geothermalis*, *D. proteolyticus*, and *D. radiodurans*). The exception is *D. maricopensis,* where two homologues are found (Deima_3154 and Deima_1878). The protein sequence alignment of these seven homologues is shown in Figure 2B and confirms the new initiation codon for a corrected DR1245 that consists of 153 amino acids (17.5 kDa). A putative domain conserved search (NCBI) indicated that the DR1245 protein group might belong to the YbjN family (pfam 10722, superfamily cl15834). Comparative genomics showed that the *D. deserti*, *D. gobiensis*, *D. geothermalis*, *D. proteolyticus dr1245* homologous genes are closely associated with a Xaa-proline dipeptidase gene encoded either right upstream the DR1245 homolog, like in *D. radiodurans* (*dr1246*) (Figure 2C), or with an intercalated LysM-domain containing gene for the other species (Figure S3). In contrast, the two *dr1245* homologous genes found in *D. maricopensis* are not directly associated with a *dr1246* homologous gene, the latter (deima_1817) being also close to a *lysM* gene (*deima_1816*). A putative oligoendopeptidase F gene is also found downstream of *DR1245* homologs from *D. deserti*, *D. gobiensis*, and *D. geothermalis* (Figure S2).

### Δ*dr1245* Bacteria are Impaired in Growth but Radioresistant

**Figure 3:**
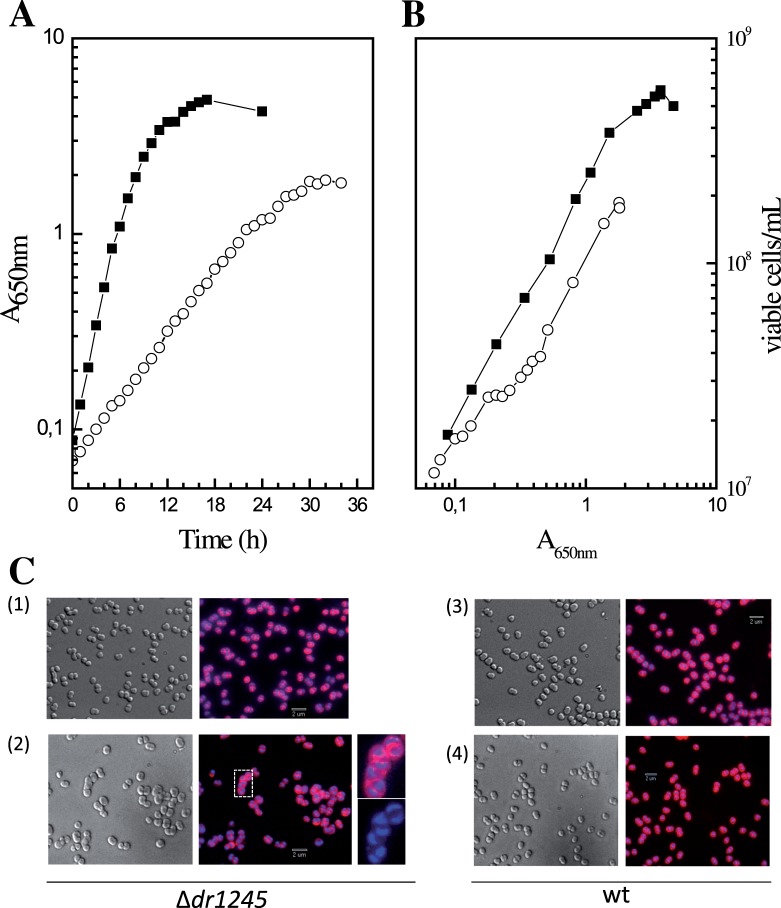
*D. radiodurans*Δ*dr1245* mutant bacteria show a growth delay and some cells are included in chains at late exponential growth phase. **A and B:** Growth of wild type and Δ*dr1245* bacteria. Wild type (close squares) and Δ*dr1245* mutant (open circles) strains were incubated at 30°C. At different times, the A_650nm_ and the numbers of viable cells per mL were measured. **C:** Cell morphology of the Δ*dr1245* bacteria at A_650nm_ = 0.27 (1) and A_650nm_ = 1.05 (2) and of wild type R1 bacteria at A_650nm_ = 0.3 (3) andA_650nm_ = 1.4 (4). Cells were observed with a wide-field three dimensional microscope. Nucleoids stained with DAPI appear in blue and membranes stained with FM-64 appear in red. Zoomed views of one indicated section showing a chain of DAPI and FM-64 stained cells or DAPI stained cells are shown.

To investigate the cellular function of DR1245, we constructed a deletion mutant in which the entire *dr1245* coding region was replaced with a *cat* cassette. Cells devoid of DR1245 protein showed an important growth defect. Indeed, the generation time of the **Δ** *dr1245* mutant growing in rich medium at 30°C was approximately three fold that of the wild type strain (300 min for the mutant versus 92 min for the wild type) (Figure 3A). The absorbance at 650 nm of **Δ** *dr1245* grown in TGY2X reached a maximum of 1.8 after 30 h incubation at 30°C versus a maximal A_650_ of 4.8 after 18 h of incubation for wild type bacteria. Moreover, at A_650_ = 1.8, the colony forming units per ml were 1.8×10^8^ for the mutant versus 4×10^8^ for the wild type indicating a slightly reduced plating efficiency of **Δ** *dr1245* mutant bacteria compared to wild type (Figure 3B). We also verified that the slow growth of the **Δ** *dr1245* mutant was due to the absence of the DR1245 protein, by showing that when *dr1245* was expressed *in trans* from the gene cloned under the control of its native promoter into plasmid p11520 [19], the **Δ** *dr1245* mutant recovered a wild type generation time (data not shown).

When **Δ** *dr1245* and wild type cells were examined by epifluorescence and deconvolution microscopy, a large proportion of **Δ** *dr1245* cells showed wild type morphology (98.5% of 1500 examined cells) at the beginning of exponential growth (A_650nm_ = 0.27) [Figure 3C (1)]. In late exponential growth (A_650nm_ = 1.05), the mutant showed an elevated number of cells included in aggregates or chains [Figure 3C (2)]. Indeed, about half of the cells examined (339 over 730) were part of small aggregates or chains containing from 6 to 20 cells, while the wild type culture was composed mainly of dyads and tetrads [Figure 3C (2)]. However, we did not observe any increase in the number of anucleate cells [Figure 3C (2)], suggesting that cells devoid of DR1245 were not impaired in DNA replication or chromosome segregation.

**Figure 4:**
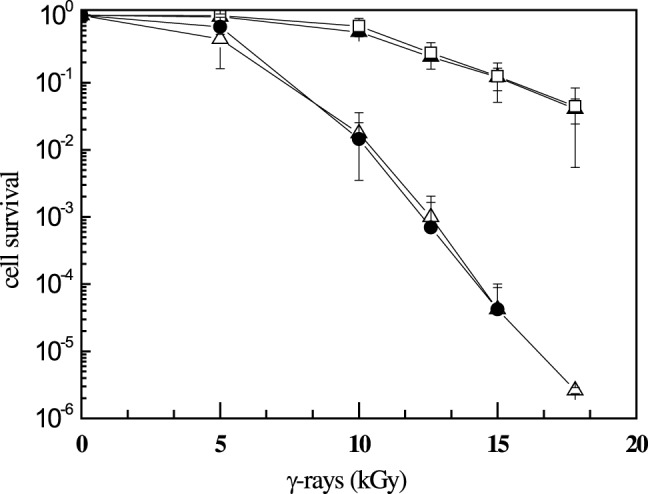
Δ*dr1245* mutant bacteria are as resistant to γ-irradiation as wild type *D. radiodurans*. Bacteria were exposed to **γ**-irradiation at doses indicated on the abscissa. Wild type (open squares), Δ*dr1245* (closed triangles), Δ*ddrB* (closed circles), Δ*ddrB* Δ*dr1245* (open triangles).

**Figure 5:**
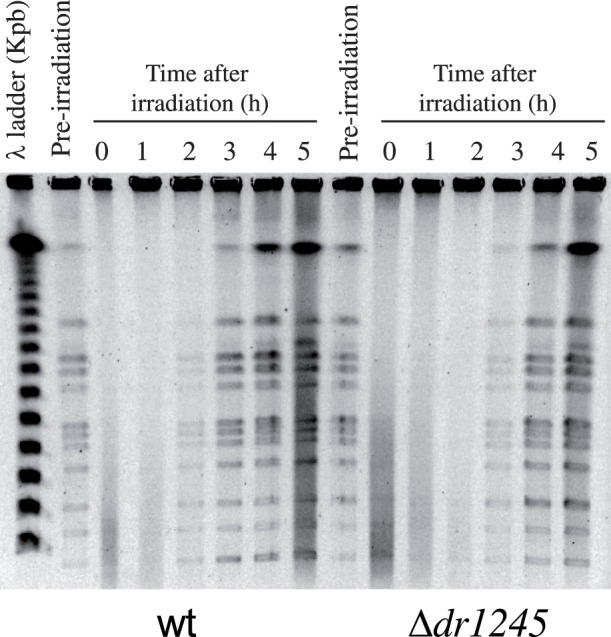
DNA double strand break repair inΔ*dr1245* mutant. Kinetics of DNA double strand break repair in wild type and Δ*dr1245* mutant followed by pulse-field gel electrophoresis (PFGE). PFGE shows *Not*I treated DNA from unirradiated cells (lane pre-irradiation) and from irradiated cells (3,800 Gy) immediately after irradiation (0) and at the indicated incubation time (hours).

To determine whether the DR1245 protein is involved in radioresistance, we compared the survival curves of **Δ** *dr1245* and the parental wild-type strain after exposure to γ-irradiation. As can be seen in Figure 4, the mutant exhibited the same radioresistance as the wild-type. Moreover the survival curve of the **Δ** *dr1245* **Δ** *ddrB* mutant can be perfectly superimposed to that of the single **Δ** *ddrB* mutant (Figure 4). To test if the absence of DR1245 has an effect on DNA double strand break repair, we measured the kinetics of DNA double-strand break repair in the **Δ** *dr1245* mutant exposed to 3,800 Gy γ-irradiation, a dose that introduces approximately 100 DNA double-strand breaks per genome equivalent in a *D. radiodurans* cell [20] but does not affect the survival of bacteria (Figure 4). Genomic DNA, extracted from the cells either immediately after irradiation or at different times during post-irradiation incubation, was analyzed by pulsed-field gel electrophoresis (Figure 5). The reconstitution of an intact genome was monitored by the appearance of a complete pattern of resolvable *Not*I fragments. Upon post-irradiation incubation, the wild-type cells reconstituted an intact genome within 2–3 hours, whereas this process took 3–4 hours in the **Δ** *dr1245* mutant (Figure 5). However, taking into account the slow growth of the **Δ** *dr1245* bacteria, these results are not sufficient to suggest a direct involvement of the DR1245 protein in DNA double strand break repair.

### DR1245 Structure

To further investigate the role of the DR1245 protein in the *D. radiodurans* bacteria, we determined the X-ray crystal structure of the protein. DR1245 crystals were obtained from a sparse-matrix screen (Qiagen). The crystallization conditions were optimized to be 1.7 M NH_4_(SO_4_)_2_, 27% w/v glycerol, which produced hexagonal rod-like crystals of DR1245 that diffracted to 1.9 Å resolution. Crystals of a Se-Met version of DR1245 failed to form. To determine the phases, native DR1245 were formed in the presence of potassium bromide and 2 Å resolution electron density maps were produced by single-wavelength anomalous dispersion (Table 1) and a molecular model was built. The asymmetric unit was composed of two monomers, which corroborates the results from the size exclusion chromatography presented above.

**Table 1:** X-ray crystallographic data collection and structure refinement statistics.

Data collection	
Space group	P6_5_
Unit cell	
a, b, c	91.71 Å, 91.71 Å, 87.75 Å
α, β, γ	90°, 90°, 120°
Wavelength	0.91983 Å
Resolution (high resolution shell)	50 - 2.0 (2.03 - 2.00) Å
Reflection measured/unique	609359/28448
Multiplicity (high resolution shell)	21.4 (15.1)
Completeness (high resolution shell)	99.59 (95.5) %
R_sym_ † (high resolution shell)	7.4 (47.0) %
* I/σ* (high resolution shell)	41.6 (4.8)
**Refinement**	
Resolution, Å	45.9–2.00 Å
R_work_/R_free_ ‡	0.164/0.195
Number of atoms	
Protein	2525
Water, ligands	255
Average B factors	
Protein atoms	20.1
Water, ligands	42.1
Root mean square deviations	
Bond lengths,	0.0112 Å
Bond angles	1.3911
Ramachandran statistics	
Residues in core region	294 (99.0%)
Residues in allowed region	3 (1%)
Residues in disallowed regions	0 (0%)

† R_sym_ = ΣΣ*j*|*Ij* -<*I*>|Σ*Ij,* where *Ij* is the intensity measurement for reflection *j* and <*I*> is the mean intensity for multiply recorded reflections.

‡ R = Σ||*F*_obs_| - |*F*_calc_||/Σ|*F*_obs_|, where the working and free R factors are calculated by using the working and free reflection sets, respectively. The free R reflections (5% of the total) were held aside throughout refinement.

**Figure 6:**
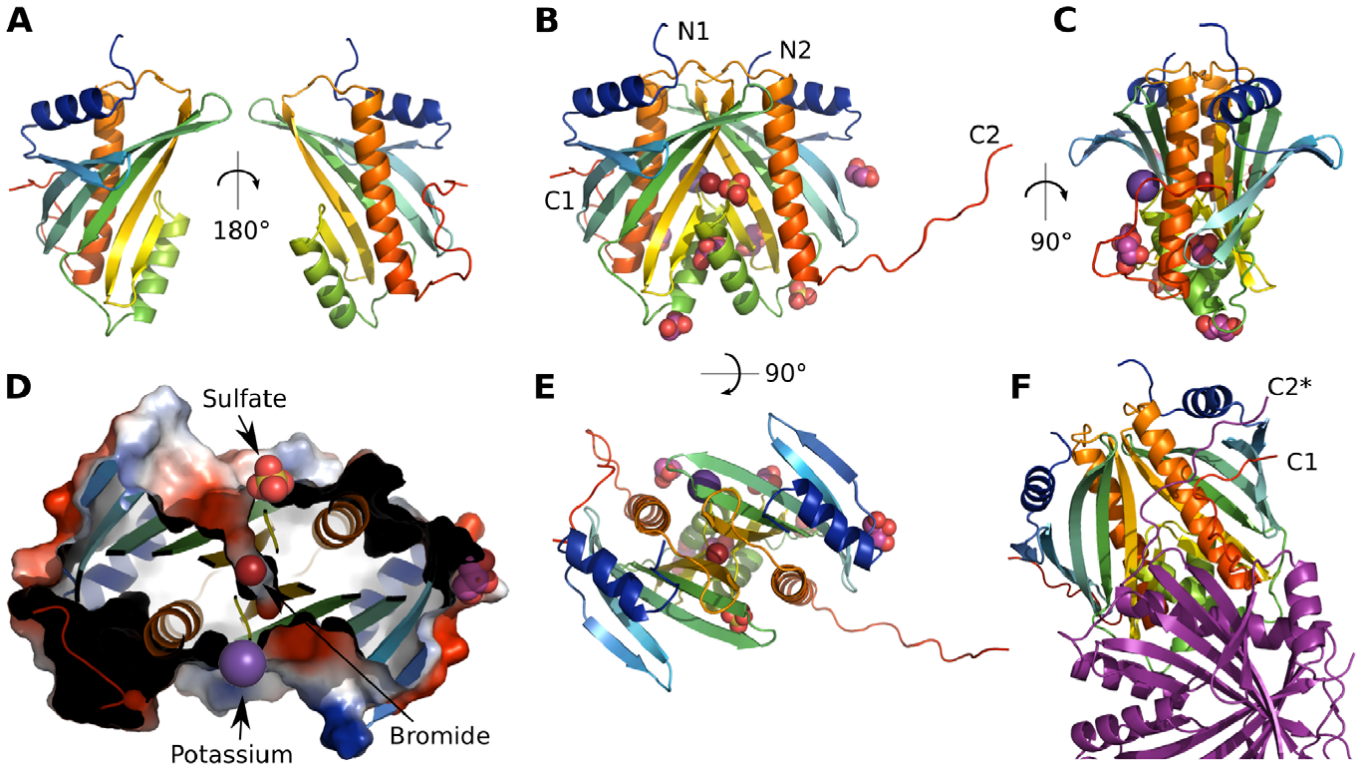
DR1245 structure. **A.** Cartoon representation of DR1245 monomer A, front and back, with chainbow colors from blue (N-terminus) to red (C-terminus). **B.** Molecules from the asymmetric unit, including a DR1245 dimer associated with two sulfates, a bromide (Red) and a potassium (Purple) ion, and five glycerol molecules (Pink). Positions of N- and C-termini are indicated. **C.** Same as in B from the side. **D.** Inside of the dimer, viewed from the bottom, represented using both cartoon and electrostatic surface, to indicate the position of the cavity that contains the bromide ion. **E.** Same as in B from the top. **F.** Two dimers as positioned in the crystal. The first dimer is represented using chain bow colors, and the other in purple. The C-terminus from the B monomer of the purple complex (C2*) interacts with the A monomer of the first complex and packs against the C-terminus of the latter (C1).

The final model includes 301 of the 306 residues from the dimer, including residues 2 to 153, and 4 to 152, of monomers A and B, respectively. Each DR1245 monomer comprises 3 α-helices packed against one side of a twisted six-stranded anti-parallel β-sheet with an α-β-β-β-α-β-β-α topology (Figure 6A). Electron density for four ions (two sulfate, one bromide, one potassium) and five glycerol molecules was observed bound to the DR1245 dimer (Figure 6B, C and E). The DR1245 dimerization interface involves residues from the β3 to β4 loop that interact with the beginning of the α3 helix of the other monomer. The α2 helix interacts with both α2 and β5 from the other monomer. Finally, the β6 sheets from both subunits interact together. The surface area buried per monomer in the dimer interface is 1,425 Å^2^ or 15.4% of the total surface area, as defined by the PISA server [21]. Interestingly, the dimerization interface harbors a cavity that contains water molecules and the bromide ion (Figure 6D). A sulfate and a potassium ion are localized at both sides of the cavity and are each coordinated by the His129 from one monomer and Arg59 from the other monomer. The two monomers are highly symmetrical except for their unstructured 11 C-terminus residues. The C-terminus from monomer A is packed against its α3 helix and β2 strand whereas the C-terminus from monomer B protrudes away to interact with the C-terminus of the A monomer from a symmetric neighboring molecule (Figure 6F). Thus, C-termini A and B from two different dimers interact together, which strongly stabilizes the crystal packing of DR1245 dimers.

### DR1245 is Structurally Similar to YbjN and Numerous Type III Secretion Chaperones

**Figure 7:**
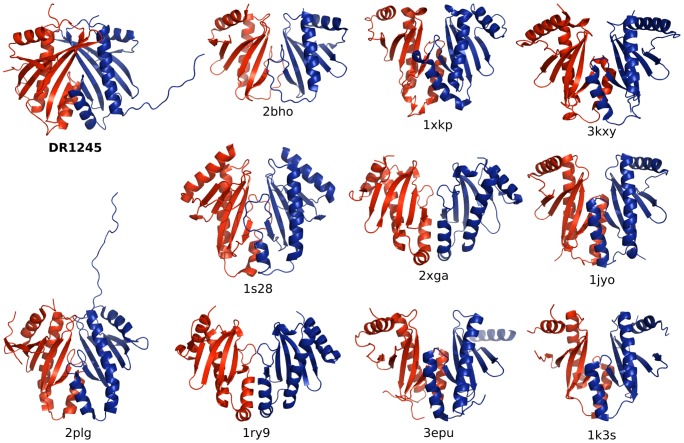
DR1245 is structurally similar to type III secretion system chaperones and YbjN homolog. Cartoon representation of DR1245 dimer with proteins from Table 2 as indicated by their PDB identification code. 2PLG is a YbjN homolog from *Synechococcus elongatus*, the other protein complexes are all type III secretion system chaperones. Monomers from each complex are represented in Red and Blue.

We performed a search for proteins that are structurally homologous to DR1245 using the DALI server [22] (Table 2). This analysis revealed a strong structural similarity between DR1245 and numerous type III secretion system (T3SS) chaperones. Structures have been reported for a number of these chaperones [23]–[27]. While these chaperones have little sequence homology, they share a common fold that is characterized by the same α-β-β-β-α-β-β-α topology as observed for DR1245 (Figure 7). Among them, only the *Synechococcus elongates* YbjN protein (PDB 2PLG) is not annotated as a T3SS chaperone. The DR1245 dimer interactions observed in the crystal structure (Figure 6F) are reminiscent to those observed with T3SS chaperones with their respective targets [23],[26], [28]–[30]. DR1245, YbjN and T3SS chaperones may thus share a common protein interaction mechanism towards their respective substrates. Thus, the DR1245 protein might have some chaperone activity towards DdrB and possibly other substrates.

**Table 2:** DR1245 presents strong structural homology to Type III secretion chaperones.

Rank	PDB	Z score	RMSD	Description
1	1S28-A	11.2	2.4	Type III secretion chaperone AvrPphF ORF1 from *Pseudomonas syringae*
				PMID: 15341731
2	2BSi-A	11.1	2.3	Type III secretion chaperone SycT from *Yersinia enterocolitica*
				PMID: 16046625
3	2BHO-A	11.1	2.4	Type III secretion chaperone SycT from *Yersinia enterocolitica*
				PMID: 16000312
4	1JYO-A	10.9	2.5	Type III secretion chaperone SicP from *Salmonella enterica*
				PMID: 11689946
5	2PLG-A	10.8	2.8	YbjN (T110839) protein from *Synechococcuselongatus*
				Putative Sensory Transduction Regulator
6	1RY9-C	10.8	2.9	Type III secretion chaperone Spa15 from *Shigellaflexneri*.
				PMID: 15088068
7	3KXY-B	10.7	2.8	Type III secretion chaperone ExsC from *Pseudomonas aeruginosa*
				PMID: 20536183
8	2XGA-B	10.7	2.8	Type III secretion chaperone Spa15 from *Shigellaflexneri*
				PMID: 21075116
9	1XKP-C	10.0	2.9	Type III secretion chaperone YscB from *Yersinia pestis*
				PMID: 15701523
10	3EPU-A	9.9	3.1	Type III secretion chaperone STM2138 from *Salmonella enterica*
11	3CXJ-D	9.8	3.1	Uncharacterized protein from *Methanothermobacterthermautotrophicus*
12	2FM8-A	9.7	3.0	Type III secretion chaperone InvB from *Salmonella enterica*
				PMID: 16507363
13	1K3S-B	9.1	2.9	Type III secretion chaperone SigE from *Salmonella enterica*
				PMID: 11685226
14	1TTW-A	8.6	2.7	Type III secretion chaperone SycH from *Yersinia Pestis*
				PMID: 15333930
15	1N5B-D	8.5	3.7	Type III secretion chaperone SycE from *Yersinia enterocolitica*
				PMID: 12554962
16	1L2W-C	8.5	3.0	Type III secretion chaperone SycE from *Yersinia pseudotuberculosis*
				PMID: 12049734
17	1JYA-A	8.4	2.9	Type III secretion chaperone SycE from *Yersinia pseudotuberculosis*
				PMID: 11685245
18	1K6Z-A	8.3	3.1	Type III secretion chaperone SycE from *Yersinia pestis*
				PMID: 11856824
19	3TU3-A	8.0	3.7	Type III secretion chaperone ExoU from *Pseudomonas aeruginosa*
20	4AKX-A	7.9	3.7	Type III secretion chaperone ExoU from *Pseudomonas aeruginosa*
				PMID: 22496657

Structural homology search using DaliLite version 3 [22] with the DR1245 chain A structure. For each PDB entry, Z-score and RMSD of the chain providing the best scores are given. When available, the PubMed identification number (PMID) of the corresponding research article is given in the Description.

## Discussion

In this work, we introduce the protein product of the *dr1245* gene of *D. radiodurans* as a DdrB partner in cells recovering from ionizing radiation (IR) treatment using the TAP-tag methodology. A direct DR1245-DdrB interaction was confirmed *in vitro* with purified proteins. Only one protein, DR1245, was found as a DdrB interactant. However, we cannot eliminate the possibility that additional proteins may interact with DdrB only when DdrB is recruited on single-stranded DNA, giving rise to transient complexes not easily purified with our TAP-tag protocol.

DR1245 has a broader action on *Deinococcus* physiology. The absence of DR1245 caused a substantial deleterious effect on the growth rate of an unstressed *Deinococcus* culture. DR1245 does not appear to have any specific function in IR resistance, and a DR1245 knockout does not affect the observed capacity of *Deinococcus* cells to recover from γ-irradiation other than to slightly slow down DNA double strand break repair. However, this DNA repair delay might result from an indirect effect: in the absence of DR1245, DNA repair could take longer simply because the cells grow more slowly.

The three-dimensional structure of DR1245 revealed its strong structural homology with type III secretion system (T3SS) chaperones and with the YbjN homolog from *Synechococcus elongatus.* Although these proteins have little sequence homology, they share a common fold that is characterized by the same α-β-β-β-α-β-β-α topology as observed for DR1245. In addition, DR1245 dimer interactions in the crystal packing are reminiscent of the way T3SS chaperones interact with their respective targets [23], [26], [28]–[30]. Type III secretion system are present in a wide range of pathogenic Gram-negative bacteria where they serve to facilitate the transport of effector proteins into infected (eukaryotic) cells through the injectisome, a needle-like multi-protein complex evolutionarily related to the bacterial flagellum (for reviews, see [31], [32]). Efficient export of the effector proteins requires specific chaperones that stabilize the effector in a partially unstructured state competent for secretion through the injectisome [33], [34]. The YbjN protein was originally identified as a multi-copy suppressor of the temperature-sensitivity conferred by point mutations in the *coaA* gene of *Escherichia coli* and has been proposed to work as a general stabilizer of some unstable proteins [35]. A recent study suggests that YbjN may play important roles in regulating cell motility, bacterial multicellular behavior, metabolism, and survival under stress conditions in *E. coli* [36]. In particular, it was shown that YbjN exhibits a negative regulatory role on exopolysaccharide production in both *E. coli* [36] and *Erwinia amylovora* [37]. The formation of chains and aggregates at late exponential phase in the absence of DR1245 might be related to an increased polysaccharide production in the *D. radiodurans* bacteria.

DR1245, YbjN and T3SS chaperones might share a common protein interaction mechanism towards their respective substrates. However, the function of this interaction might differ between these “chaperones”. Interestingly, the *dr1245* homologous genes are often closely associated to a gene encoding a Xaa-proline dipeptidase located either right upstream *dr1245*, like in *D. radiodurans*, or with an intercalated LysM-domain containing gene. A putative oligoendopeptidase F gene is also found downstream *DR1245* homologs from *D. deserti*, *D. gobiensis*, and *D. geothermalis*. These peptidases may work in concert with DR1245 in a protein degradation system involved in the regulation of the lifetime of many proteins in the *Deinococcus* proteome, accounting for the broader action of DR1245 on *Deinococcus* physiology.

In conclusion, in an effort to find DdrB partners, we identified the DR1245 protein, a protein specific to the *Deinococcaceae* with unknown function. In spite of the involvement of DdrB in DNA double strand break repair in *D. radiodurans*, DR1245 does not seem to be implicated in the recovery process after ionizing radiation treatment. DR1245 structure is reminiscent of those of YbjN and T3SS secretion chaperons. A putative DR1245 chaperone activity has to be further investigated, in particular to find other DR1245 cellular targets to explain the dramatic effect on cell growth of the absence of the DR1245 protein and to elucidate the precise functions of the DR1245 protein.

## Materials and Methods

This work was carried out in compliance with the current laws governing genetic experimentation in France and USA.

### Bacterial Strains, Plasmids, Oligonucleotides, Media

Bacterial strains and plasmids are listed in Table 3 and Table 4, respectively. The *Escherichia coli* strains used were DH5α as the general cloning host, strain SCS110 to propagate plasmids prior to introduction into *D. radiodurans* via transformation [38] and the BL21(DE3) strain (Stratagene) to express proteins before purification. All *D. radiodurans* strains were derivatives of the wild-type strain R1 ATCC 13939. Alleles Δ*dr1245*Ω*cat* and *rpoB*::*spa*Ω*cat* were constructed by the tripartite ligation method [39]. The genetic structure and the purity of the mutants were checked by PCR. Oligonucleotides used for strain construction and diagnostic PCR will be provided on request. Transformation of *D. radiodurans* with PCR products, genomic DNA, or plasmids was performed as previously described [14].

**Table 3:** Bacterial strains.

Bacterial strains	Genotype	Source or reference
*E. coli*		
**DH5α**	*supE44*Δ*lacU(φ80lacZ*Δ*M15) hsdR17 recA1 endA1 gyrA96 thi-1 relA1*	laboratory stock
**SCS110**	*endA dam dcm supE44*Δ(*lac*-*proAB*) (*F’traD36 proAB lacI^q^Z*Δ*M15*)	laboratory stock
**BL21(DE3)**	*F– ompT gal dcm lon hsdS_B_(r_B_- m_B_-) λ(DE3 [lacI lacUV5-T7 gene 1 ind1 sam7 nin5])*	Stratagen
*D. radiodurans*		
**R1**	ATCC 13939	laboratory stock
**GY12809**	*rpoB*::*spa*Ω*cat*	this work
**GY12830**	*ddrB*::*spa*Ω*cat*	[14]
**GY12834**	Δ*dr1245*Ω*cat*	this work
**GY12835**	Δ*ddrB*Ω*kan*	[14]
**GY12837**	Δ*ddrB*Ω*kan*Δ*dr1245*Ω*cat*	this work
**GY12844**	**Δ** *dr1245*Ω*cat/*p12765	this work
**GY12845**	Δ*dr1245*Ω*cat/*p11520	this work

**Table 4:** Plasmids used.

Plasmids	Description	Source
p11086	source of kanamycin cassette in *D. radiodurans*	laboratorystock
p12723	source of spa-tag chloramphenicol cassette	[48]
p11520	Shuttle vector Spec^R^ in *E. coli* and in *D. radiodurans*	[19]
p12764	source of HA-tag kanamycin cassette	[49]
p12765	Derived from p11520 by cloning *dr1245* gene	this work
pET21d	expression vector	Novagen
pEAW562	pET21d with a PCR fragment encoding *DR1245*	this work

*D. radiodurans* strains were grown at 30°C in TGY2x (1% tryptone, 0.2% dextrose, 0.6% yeast extract) or plated on TGY1x containing 1,5% agar and *E. coli* strains were grown at 37°C in Lysogeny Broth. When necessary, media were supplemented with the appropriate antibiotics used at the following final concentrations: kanamycin, 6 µg/mL; chloramphenicol, 3 µg/mL; spectinomycin, 75 µg/mL for *D. radiodurans* and 40 µg/mL for *E. coli*; and ampicillin, 100 µg/mL.

### DNA Manipulations

Plasmid DNA was extracted from *E. coli* using the QIAprep spin miniprep kit (Qiagen). Chromosomal DNA of *D. radiodurans* was isolated from stationary phase cells in TGY2x medium. 2 mL cultures were harvested by centrifugation (9,000 *g* at room temperature). Pellets were resuspended in 100 µL of lysis buffer (2% Triton-X 100, 1% SDS, 100 mM NaCl, 1 mM EDTA) and disrupted with a fastprep desintegrator (Savant; Bio101) using 0.1 g of glass beads (500 µM) in the presence of 100 µL of phenol-chloroform for 120 s. 200 µL of SSC 1X (150 mM NaCl; 15 mM sodium citrate) was added. After centrifugation for 3 min at 9,000 *g*, supernatants were treated with 1 volume of phenol-chloroform-isoamylacohol (24∶1 v/v). DNA was precipitated with ethanol and resuspended in 50 µL of TE (10 mM Tris-Cl; 1 mM EDTA) plus 4 µL of RNase (5 mg/mL). Amplification of plasmid or genomic DNA by PCR was performed with Phusion DNA polymerase (Thermo Scientific) or Go Taq DNA polymerase (Promega).

### TAP Purification of Protein Complexes

*D. radiodurans* strains in which a bait protein had been tagged at their C-terminal end with a SPA motif were grown in 1 liter of TGY2x medium supplemented with chloramphenicol until A_650nm_ = 2.2. When necessary, cells were concentrated to an A_650nm_ = 20 in TGY2x and irradiated on ice at 3,800 Gy with a ^137^Cs irradiation system at a dose rate of 41.8 Gy/min (Institut Curie, Orsay, France). Following irradiation, cultures were diluted in TGY2x to an A_650nm_ = 0.45 and incubated at 30°C for 45 min. Cells were harvested (15 min, 6,000 *g* at 4°C) and pellets were washed with 100 mL of buffer A (10 mM Tris-Cl pH 7.5, 150 mM NaCl). Cells were centrifuged and pellets were frozen at -80°C until purification.

Pellets, resuspended in 15 mL of buffer B (10 mM Tris-Cl pH 7.5, 150 mM NaCl, 0.5 mM DTT, 0.2 mM EDTA) containing 10 mg/mL of lysozyme, were incubated at 37°C for 40 min and immediately frozen in liquid nitrogen. Cells were disrupted with a Bead Beater instrument (Biospec products ) using 0.8 g of zirconia beads (100 µm) and 2 pulses of 2 min. Cell debris were removed by centrifugation at 20,000 *g* for 30 min at 4°C and the supernatant constituted the cell extract. Supernatants were mixed with 200 µL of pre-equilibrated anti-FLAG M2 resin (Sigma) in 2 mL tubes, which were placed on a rotating wheel for 3 h30 at 4°C. After washing with 10 mL of M2 buffer (10 mM TrisCl, pH 7.5, 150 mM NaCl, Triton- X 100 0.1%), the resin, after centrifugation (5 min, 4,000 *g*) was resuspended in 200 µL of TEV buffer (50 mM Tris-Cl, pH 7.5, NaCl 150 mM, EDTA 0.2 mM, Triton-X 100 0.1%, 1 mM DTT) with 50 units of AcTEV protease (Invitrogen). TEV cleavage was performed overnight with rotation at 4°C. The anti-FLAG M2 resin was washed with 400 µL of buffer TEV containing 2 mM CaCl_2_ and the eluate was collected and mixed with 100 µL Calmodulin Sepharose resin (GE Healthcare) pre-equilibrated with CBB buffer (10 mM Tris-Cl, pH 7.5, 150 mM NaCl, 10 mM β-mercaptoethanol, 2 mM CaCl_2_). After incubation under gentle agitation for 2 h30 at 4°C, the resin was washed twice with 250 µL of CBB buffer and once with 100 µL of CWB buffer (10 mM Tris-Cl, pH 7.5, 150 mM NaCl, 10 mM β-mercaptoethanol, 0.1 mM CaCl_2_). The proteins remaining bound to the calmodulin resin were eluted with 400 µL of CEB buffer (50 mM Tris-Cl, pH 7.5, 150 mM NaCl, 10 mM β-mercaptoethanol, 3 mM EGTA). Proteins from the eluate were precipitated overnight at −20°C by the addition of three volumes of acetone. After centrifugation at 15,000 *g* for 30 min at 4°C, acetone was discarded and the pellets were dried out to analyze their protein contents by mass spectrometry.

### Protein Identification by Tandem Mass Spectrometry

Protein spots were excised from SDS-PAGE gels and treated as previously described [40]. They were trypsin digested and the resulting peptides were first analysed with an Esquire 3000 Plus ion trap mass spectrometer (Brucker Daltonics), and then further with a LTQ-Orbitrap XL hybrid mass spectrometer (ThermoFisher) coupled to an UltiMate 3000 LC system (Dionex-LC Packings). The latter system was operated as described previously [41]. Peptide mixtures (5 µL) were loaded and desalted online on a reverse phase precolumn C18 Pepmap 100 column (LC Packings). Then, they were resolved on a nanoscale C18 Pepmap 100™ capillary column (LC Packings) at a flow rate of 0.3 µL per min with a gradient of CH_3_CN/0.1% formic acid prior to injection into the mass spectrometer. Peptides were separated using a 90 min gradient from 5 to 60% solvent B (0.1% HCOOH/80% CH_3_CN). Solvent A was 0.1% HCOOH/100% H_2_O. The full-scan mass spectra were measured from *m/z* 300 to 2,000 with the LTQ-orbitrap XL mass spectrometer operated in the data-dependent mode using the TOP3 strategy. In brief, a scan cycle was initiated with a full scan of high mass accuracy in the Orbitrap, which was followed by MS/MS scans in the linear ion trap on the three most abundant precursor ions with dynamic exclusion of previously selected ions. MS/MS spectra were assigned with the MASCOT 2.2 software (Matrix Science) using the NCBI nr protein sequence database or a home-made database containing the DR1245 corrected sequence. Peptide tolerance was set at 5 ppm, MS/MS tolerance at 0.4 Da, peptide charge at +1/+2/+3, static modification of carboxy amido methylated Cys (+57.0215), dynamic modification of oxidized Met (+15.9949), and maximum number of missed cleavage at 1. Only peptides on rank 1 with a p value below 0.05 were selected. Proteins identified by at least 2 different peptides were validated.

### Survival Curve

Exponential cultures, grown in TGY2x (supplemented with spectinomycin when necessary), were concentrated to an A_650nm_ = 20 in TGY2x and irradiated on ice with a ^137^Cs irradiation system (Institut Curie, Orsay, France) at a dose rate of 41.8 Gy/min. Following irradiation, diluted samples were plated on TGY plates. Colonies were counted after 3–4 days incubation at 30°C.

### Fluorescence Microscopy

Cells were grown in TGY2x to an A_650nm_ = 0.5. Aliquots (1 mL) were removed and the cells were fixed using toluene at 3% final concentration. Cell membranes were stained with N-(3-triethylammonium-propyl)-4-(6-(4-(diethylamino)phenyl)hexatrienyl)pyridinium dibromide (FM 4–64) at 10 µg/mL and the nucleoid with 4,6-diamidino-2-phenylindole dihydrochloride (DAPI) at 2 µg/mL. FM 4–64 stains the lipid membranes with red fluorescence (excitation/emission 515/640 nm) and DAPI stains the nucleoid with blue fluorescence (excitation/emission 350/470 nm). The stained cells were observed using a Leica DM RXA microscope. Images were captured with a CDD camera 5 MHz Micromax 1300Y (Roper Instruments). The final reconstructed images were obtained by deconvoluting Z-series with metamorph software (Universal Imaging).

### Kinetics of DNA Repair Measured by Pulse-field Gel Electrophoresis

Non-irradiated or irradiated (3,800 Gy) cultures were diluted in TGY2x to an A_650nm_ = 0.2 and incubated at 30°C. At different post-irradiation recovery times, culture aliquots (5 mL) were removed to prepare DNA plugs as described previously [10]. The agarose embedded DNA plugs were digested for 16 h at 37°C with 10 units of *Not*I restriction enzyme. After digestion, the plugs were subjected to pulsed field gel electrophoresis as described previously [10].

### Expression and Purification of *D. radiodurans* DR1245

The reannotated DR1245 gene was PCR amplified from *D. radiodurans* R1 genomic DNA, and cloned in pET21d (Novagen) at *Nco*I and *Pst*I sites to yield construct pEAW562. The resulting construct was designed to overexpress the native form of DR1245. The construct was inserted in the BL21(DE3) strain (Stratagene) and the resulting cells were grown in 10 liters LB broth containing 100 µg/mL ampicillin at 37°C to an A_600nm_ of 0.5. Overexpression of DR1245 was then induced with 0.4 mM IPTG (GoldBio) and cells were grown at 37°C for three more hours before harvest. The 23 g cell pellet was frozen in liquid nitrogen and thawed overnight at 4°C in 5 volumes of 250 mM Tris-Cl, pH 8, 25% w/v sucrose. All subsequent steps were performed at 4°C. Lyzozyme (Sigma) and EDTA were added to a final concentration of 0.2 mg/mL and 7 mM respectively. Cells were stirred for 2 h and then sonicated on ice. Insoluble material and cell debris were pelleted and removed by centrifugation at 38,000 *g* for 1 h. 25 mL of 5% polyethylenimine solution, pH 7.5, were added drop-wise to the 200 mL cell lysate supernatant and the resulting solution was centrifuged 15 min at 9,000 *g*. The supernatant was discarded and proteins, including DR1245, contained in the resulting pellet were eluted from the pellet with R buffer (20 mM Tris-Cl 80% cations, 100 µM EDTA, 1 mM DTT, and 10% w/v glycerol) +300 mM NH_4_(SO_4_)_2_ (MP Biochemical). The resulting suspension was centrifuged 15 min at 9,000 *g*. The supernatant was recovered and brought to 40% saturation by additional NH_4_(SO_4_)_2_. The solution was stirred overnight and centrifuged 1 h at 25,000 *g*. The DR1245 protein remained in the pellet and was eluted from the pellet using R buffer containing 1 M NH_4_(SO_4_)_2_. The resuspended protein was then loaded on a Butyl Sepharose (Amersham) column (bed volume equivalent to volume used to resuspend the cell pellet) using an AKTA FPLC system. DR1245 bound to the resin and was eluted as a broad peak in the late gradient from R buffer containing 1 M NH_4_(SO_4_)_2_ to R buffer alone. The corresponding fractions were pooled and dialyzed against P buffer (20 mM KPO_4_, pH 7.5, 0.1 mM EDTA, 1 mM DTT, and 10% w/v glycerol) in order to be loaded on a 6 mL hydroxyapatite column (Biorad), from which DR1245 flowed through, and similarly on 1 mL sepharose SP, CM and heparin columns (Amersham) that were attached together. DR1245 flowed through all these columns and was loaded on a 23 mL DEAE sepharose column. DR1245 bound to the DEAE resin and eluted in the gradient to buffer P containing 300 mM phosphate. As some high molecular weight contaminants remained, the protein solution was finally loaded on a Sephacryl S-300 16/60 HR (Amersham Biosciences) size exclusion column equilibrated with R buffer. Fractions containing >99% pure protein (as estimated from SDS-PAGE) were pooled, aliquoted, snap frozen in liquid nitrogen, and stored at −80°C. The purified protein was free of any detectable nuclease activity. DR1245 concentrations were estimated using ε_280nm_ = 19,060 M^−1^cm^−1^ and 1.0885 (mg/mL)^−1^cm^−1^ as extinction coefficients.

### Immunopulldowns

The whole experiment was performed at room temperature (21°C). Streptavidin agarose beads (Thermo) were saturated with biotinylated anti-DdrB affinity purified chicken polyclonal IgY antibody (GeneTel Laboratories, LLC, Madison) in 1x PBS-Tween (10 mM sodium phosphate, 150 mM NaCl, 0.05% Tween-20, pH 7.5) and incubated for 1 hour with agitation. The slurry was then washed with 1x PBS-Tween to remove excess antibody. For each assay, 10 nmol purified native DdrB was then mixed with 200 µL (50% v/v) antibody bead slurry and the mixture was incubated for 1 hour with agitation. The slurry was then applied to plastic capped mini-columns that fit 2 mL microcentrifuge tubes and washed with 5 times 500 µL 1x PBS-Tween to remove excess DdrB by centrifugation at 500 *g*. 10 nmol of DR1245 or of *D. radiodurans* SSB [as purified in [42]] was then applied to the slurry and incubated for 1 hour with agitation. The flowthrough was recovered by centrifugation at 500 *g* and kept aside. The resin was then washed using 6 times 500 µL 1x PBS-Tween. Proteins remaining on the resin were eluted using 10x concentrated PBS-Tween. Flowthrough and elution fractions were concentrated using TCA precipitation and loaded on a 12% SDS-PAGE. Following migration, the gel was revealed by Coomassie staining using standard procedures. Control assays indicated that none of the tested proteins interacted with the Streptavidin agarose beads or biotinylated anti-DdrB IgY.

### DR1245 Crystal Structure Determination

Crystals of native bromine-bound DR1245 were grown by hanging drop vapor diffusion by mixing equal volumes of DR1245 (20 mg/mL in 20 mM Tris-Cl, pH 8, 100 µM EDTA, 1 mM DTT, and 10% w/v glycerol) with mother liquor (1.7 M NH_4_(SO_4_)_2_, 27% w/v glycerol, 100 mM KBr) and equilibration at room temperature. Crystals appeared within 2 days and were directly frozen in liquid nitrogen without additional cryoprotection. Diffraction data, collected at the Advanced Photon Source (Argonne National Laboratory) LS-CAT beamline station 21-ID-D, were indexed and scaled using HKL2000 [43]. The DR1245 structure was determined using the single-wavelength anomalous dispersion phasing method by taking advantage of the anomalous signal from bound Br atoms. The location of Br atom positions and initial phase calculations were performed using the Phenix program suite [44] and an initial map was obtained with Autorickshaw [45]. Finally, iterative rounds of model building and structure refinement were carried out using Coot [46] and REFMAC [47], respectively. Model quality was assessed using Rampage, Procheck and Sfcheck from the CCP4 program suite [47]. X-ray crystallographic data collection and structure refinement statistics are presented in Table 1. Atomic coordinates and structure factors for the DR1245 structure were deposited in the Protein Data Bank with PDB-code 4H5B.

## Supporting Information

Figure S1 **Purification of RpoB partners.** SDS-PAGE analysis of protein complexes purified from GY12809 strain. The tagged protein is RpoB and purification was carried out from exponential cells. * This band corresponds to the tagged RpoB fused with the calmodulin binding protein.
(PDF)

Figure S2 **MS/MS spectrum of the peptide [METALLTLDTLAK] corresponding to the N-terminus of DR1245 protein.** The mass of the parent di-charged ion was measured at *m/z* 718.3895 (mass error 0.38 ppm) with an LTQ-Orbitrap XL mass spectrometer (Thermo). The annotated secondary *b* and *y* ions are indicated.
(PDF)

Figure S3 **Genome context of** ***deinococcal dr1245*** **homologous genes.** *dr1245* homologues from sequenced Deinococcacae (*D. radiodurans*, *dr1245*; *D. gobiensis*, *dgo_CA1600*, *D. deserti*, *deide_11730*; *D. geothermalis*, *dgeo_1085*; *D. proteolyticus*, *deipr_0969*) were colored in dark blue whereas, the neighbouring proline dipeptidase, *lysM*, and, oligoendopeptidase F genes were colored in orange, grey and yellow, respectively.
(PDF)

Table S1 **List of peptides identified by tandem mass spectrometry and their characteristics.**
(XLSX)

## References

[1] Cox MM, Battista JR (2005) *Deinococcus radiodurans* - the consummate survivor. Nat Rev Microbiol 3: 882–892. 16261171 10.1038/nrmicro1264

[2] Blasius M, Hubscher U, Sommer S (2008) *Deinococcus radiodurans*: what belongs to the survival kit? Crit Rev Biochem Mol Biol 43: 221–238. 18568848 10.1080/10409230802122274

[3] Slade D, Radman M (2011) Oxidative Stress Resistance in *Deinococcus radiodurans* . Microbiol Mol Biol Rev 75: 133–191. 21372322 10.1128/MMBR.00015-10 PMC3063356

[4] Daly MJ, Gaidamakova EK, Matrosova VY, Vasilenko A, Zhai M, et al (2007) Protein oxidation implicated as the primary determinant of bacterial radioresistance. PLoS Biol 5: e92. 17373858 10.1371/journal.pbio.0050092 PMC1828145

[5] Daly MJ (2009) A new perspective on radiation resistance based on *Deinococcus radiodurans* . Nat Rev Microbiol 7: 237–245. 19172147 10.1038/nrmicro2073

[6] Krisko A, Radman M (2010) Protein damage and death by radiation in *Escherichia coli* and *Deinococcus radiodurans* . Proc Natl Acad Sci U S A 107: 14373–14377. 20660760 10.1073/pnas.1009312107 PMC2922536

[7] Daly MJ (2012) Death by protein damage in irradiated cells. DNA Repair (Amst) 11: 12–21. 22112864 10.1016/j.dnarep.2011.10.024

[8] Liu Y, Zhou J, Omelchenko MV, Beliaev AS, Venkateswaran A, et al (2003) Transcriptome dynamics of *Deinococcus radiodurans* recovering from ionizing radiation. Proc Natl Acad Sci U S A 100: 4191–4196. 12651953 10.1073/pnas.0630387100 PMC153069

[9] Tanaka M, Earl AM, Howell HA, Park MJ, Eisen JA, et al (2004) Analysis of *Deinococcus radiodurans*’s transcriptional response to ionizing radiation and desiccation reveals novel proteins that contribute to extreme radioresistance. Genetics 168: 21–33. 15454524 10.1534/genetics.104.029249 PMC1448114

[10] Harris DR, Tanaka M, Saveliev SV, Jolivet E, Earl AM, et al (2004) Preserving genome integrity: the DdrA protein of *Deinococcus radiodurans* R1. PLoS Biol 2: e304. 15361932 10.1371/journal.pbio.0020304 PMC515370

[11] Narumi I, Satoh K, Cui S, Funayama T, Kitayama S, et al (2004) PprA: a novel protein from *Deinococcus radiodurans* that stimulates DNA ligation. Mol Microbiol 54: 278–285. 15458422 10.1111/j.1365-2958.2004.04272.x

[12] Jolivet E, Lecointe F, Coste G, Satoh K, Narumi I, et al (2006) Limited concentration of RecA delays DNA double-strand break repair in Deinococcus radiodurans R1. Mol Microbiol 59: 338–349. 16359339 10.1111/j.1365-2958.2005.04946.x

[13] Xu G, Lu H, Wang L, Chen H, Xu Z, et al (2010) DdrB stimulates single-stranded DNA annealing and facilitates RecA-independent DNA repair in *Deinococcus radiodurans* . DNA Repair (Amst) 9: 805–812. 20451472 10.1016/j.dnarep.2010.04.006

[14] Bouthier de la Tour C, Boisnard S, Norais C, Toueille M, Bentchikou E, et al (2011) The deinococcal DdrB protein is involved in an early step of DNA double strand break repair and in plasmid transformation through its single-strand annealing activity. DNA Repair (Amst) 10: 1223–1231. 21968057 10.1016/j.dnarep.2011.09.010 PMC3268515

[15] Norais CA, Chitteni-Pattu S, Wood EA, Inman RB, Cox MM (2009) DdrB protein, an alternative *Deinococcus radiodurans* SSB induced by ionizing radiation. J Biol Chem 284: 21402–21411. 19515845 10.1074/jbc.M109.010454 PMC2755865

[16] Sugiman-Marangos S, Junop MS (2010) The structure of DdrB from *Deinococcus*: a new fold for single-stranded DNA binding proteins. Nucleic Acids Res 38: 3432–3440. 20129942 10.1093/nar/gkq036 PMC2879517

[17] Zeghouf M, Li J, Butland G, Borkowska A, Canadien V, et al (2004) Sequential Peptide Affinity (SPA) system for the identification of mammalian and bacterial protein complexes. J Proteome Res 3: 463–468. 15253427 10.1021/pr034084x

[18] White O, Eisen JA, Heidelberg JF, Hickey EK, Peterson JD, et al (1999) Genome sequence of the radioresistant bacterium *Deinococcus radiodurans* R1. Science 286: 1571–1577. 10567266 10.1126/science.286.5444.1571 PMC4147723

[19] Bentchikou E, Servant P, Coste G, Sommer S (2007) Additive effects of SbcCD and PolX deficiencies in the *in vivo* repair of DNA double strand breaks in *Deinococcus radiodurans* . J Bacteriol 189: 4787–4790. 10.1128/JB.00452-07 PMC1913444 17483232

[20] Battista JR (1997) Against all odds: the survival strategies of *Deinococcus radiodurans* . Annu Rev Microbiol 51: 203–224. 9343349 10.1146/annurev.micro.51.1.203

[21] Krissinel E, Henrick K (2007) Inference of macromolecular assemblies from crystalline state. J Mol Biol 372: 774–797. 17681537 10.1016/j.jmb.2007.05.022

[22] Holm L, Rosenstrom P (2010) Dali server: conservation mapping in 3D. Nucleic Acids Res 38: W545–549. 20457744 10.1093/nar/gkq366 PMC2896194

[23] Stebbins CE, Galan JE (2001) Maintenance of an unfolded polypeptide by a cognate chaperone in bacterial type III secretion. Nature 414: 77–81. 11689946 10.1038/35102073

[24] Locher M, Lehnert B, Krauss K, Heesemann J, Groll M, et al (2005) Crystal structure of the *Yersinia enterocolitica* type III secretion chaperone SycT. J Biol Chem 280: 31149–31155. 16000312 10.1074/jbc.M500603200

[25] Buttner CR, Cornelis GR, Heinz DW, Niemann HH (2005) Crystal structure of *Yersinia enterocolitica* type III secretion chaperone SycT. Protein Sci 14: 1993–2002. 16046625 10.1110/ps.051474605 PMC2279310

[26] Vogelaar NJ, Jing X, Robinson HH, Schubot FD (2010) Analysis of the crystal structure of the ExsC.ExsE complex reveals distinctive binding interactions of the *Pseudomonas aeruginosa* type III secretion chaperone ExsC with ExsE and ExsD. Biochemistry 49: 5870–5879. 20536183 10.1021/bi100432e

[27] Schreiner M, Niemann HH (2012) Crystal structure of the *Yersinia enterocolitica* type III secretion chaperone SycD in complex with a peptide of the minor translocator YopD. BMC Struct Biol 12: 13. 22708907 10.1186/1472-6807-12-13 PMC3443056

[28] Birtalan SC, Phillips RM, Ghosh P (2002) Three-dimensional secretion signals in chaperone-effector complexes of bacterial pathogens. Mol Cell 9: 971–980. 12049734 10.1016/s1097-2765(02)00529-4

[29] Phan J, Tropea JE, Waugh DS (2004) Structure of the *Yersinia pestis* type III secretion chaperone SycH in complex with a stable fragment of YscM2. Acta Crystallogr D Biol Crystallogr 60: 1591–1599. 15333930 10.1107/S0907444904017597

[30] Lilic M, Vujanac M, Stebbins CE (2006) A common structural motif in the binding of virulence factors to bacterial secretion chaperones. Mol Cell 21: 653–664. 16507363 10.1016/j.molcel.2006.01.026

[31] Wilharm G, Dittmann S, Schmid A, Heesemann J (2007) On the role of specific chaperones, the specific ATPase, and the proton motive force in type III secretion. Int J Med Microbiol 297: 27–36. 17126597 10.1016/j.ijmm.2006.10.003

[32] Izore T, Job V, Dessen A (2011) Biogenesis, regulation, and targeting of the type III secretion system. Structure 19: 603–612. 21565695 10.1016/j.str.2011.03.015

[33] Page AL, Parsot C (2002) Chaperones of the type III secretion pathway: jacks of all trades. Mol Microbiol 46: 1–11. 12366826 10.1046/j.1365-2958.2002.03138.x

[34] Feldman MF, Cornelis GR (2003) The multitalented type III chaperones: all you can do with 15 kDa. FEMS Microbiol Lett 219: 151–158. 12620614 10.1016/S0378-1097(03)00042-9

[35] Chen X, Shen D, Zhou B (2006) Analysis of the temperature-sensitive mutation of *Escherichia coli* pantothenate kinase reveals YbjN as a possible protein stabilizer. Biochem Biophys Res Commun 345: 834–842. 16701556 10.1016/j.bbrc.2006.04.101

[36] Wang D, Calla B, Vimolmangkang S, Wu X, Korban SS, et al (2011) The orphan gene *ybjN* conveys pleiotropic effects on multicellular behavior and survival of *Escherichia coli* . PLoS One 6: e25293. 21980417 10.1371/journal.pone.0025293 PMC3181261

[37] Wang D, Korban SS, Pusey PL, Zhao Y (2012) AmyR Is a Novel Negative Regulator of Amylovoran Production in *Erwinia amylovora* . PLoS One 7: e45038. 23028751 10.1371/journal.pone.0045038 PMC3445560

[38] Meima R, Rothfuss HM, Gewin L, Lidstrom ME (2001) Promoter cloning in the radioresistant bacterium *Deinococcus radiodurans* . J Bacteriol 183: 3169–3175. 11325946 10.1128/JB.183.10.3169-3175.2001 PMC95218

[39] Mennecier S, Coste G, Servant P, Bailone A, Sommer S (2004) Mismatch repair ensures fidelity of replication and recombination in the radioresistant organism *Deinococcus radiodurans* . Mol Genet Genomics 272: 460–469. 15503140 10.1007/s00438-004-1077-6

[40] Gabant G, Augier J, Armengaud J (2008) Assessment of solvent residues accessibility using three Sulfo-NHS-biotin reagents in parallel: application to footprint changes of a methyltransferase upon binding its substrate. J Mass Spectrom 43: 360–370. 17968972 10.1002/jms.1328

[41] Clair G, Roussi S, Armengaud J, Duport C (2010) Expanding the known repertoire of virulence factors produced by *Bacillus cereus* through early secretome profiling in three redox conditions. Mol Cell Proteomics 9: 1486–1498. 20368289 10.1074/mcp.M000027-MCP201 PMC2938089

[42] Eggington JM, Haruta N, Wood EA, Cox MM (2004) The single-stranded DNA-binding protein of *Deinococcus radiodurans* . BMC Microbiol 4: 2. 14718065 10.1186/1471-2180-4-2 PMC331404

[43] Otwinowski Z, Minor W (1997) Processing of X-ray diffraction data collected in oscillation mode. Methods in Enzymology. 307–326. 10.1016/S0076-6879(97)76066-X 27754618

[44] Adams PD, Afonine PV, Bunkoczi G, Chen VB, Davis IW, et al (2010) PHENIX: a comprehensive Python-based system for macromolecular structure solution. Acta Crystallogr D Biol Crystallogr 66: 213–221. 20124702 10.1107/S0907444909052925 PMC2815670

[45] Panjikar S, Parthasarathy V, Lamzin VS, Weiss MS, Tucker PA (2005) Auto-rickshaw: an automated crystal structure determination platform as an efficient tool for the validation of an X-ray diffraction experiment. Acta Crystallogr D Biol Crystallogr 61: 449–457. 15805600 10.1107/S0907444905001307

[46] Emsley P, Cowtan K (2004) Coot: model-building tools for molecular graphics. Acta Crystallogr D Biol Crystallogr 60: 2126–2132. 15572765 10.1107/S0907444904019158

[47] Dodson EJ, Win M, Ralph A (1997) Collaborative Computational Project, number 4: providing programs for protein crystallography. Methods in Enzymology. 620–633. 10.1016/s0076-6879(97)77034-4 18488327

[48] Bouthier de la Tour C, Toueille M, Jolivet E, Nguyen HH, Servant P, et al (2009) The *Deinococcus radiodurans* SMC protein is dispensable for cell viability yet plays a role in DNA folding. Extremophiles 13: 827–837. 19629621 10.1007/s00792-009-0270-2

[49] Toueille M, Mirabella B, Guerin P, Bouthier de la Tour C, Boisnard S, et al (2012) A comparative proteomic approach to better define *Deinococcus* nucleoid specificities. J Proteomics 75: 2588–2600. 22446890 10.1016/j.jprot.2012.03.002

