# Supplementary figures and images for: The Deinococcus radiodurans DR1245 Protein, a DdrB Partner Homologous to YbjN Proteins and Reminiscent of Type III Secretion System Chaperones
Source: PLoS One. 2013 Feb 18;8(2):e56558. doi: 10.1371/journal.pone.0056558 (PMC3575483; doi:10.1371/journal.pone.0056558)

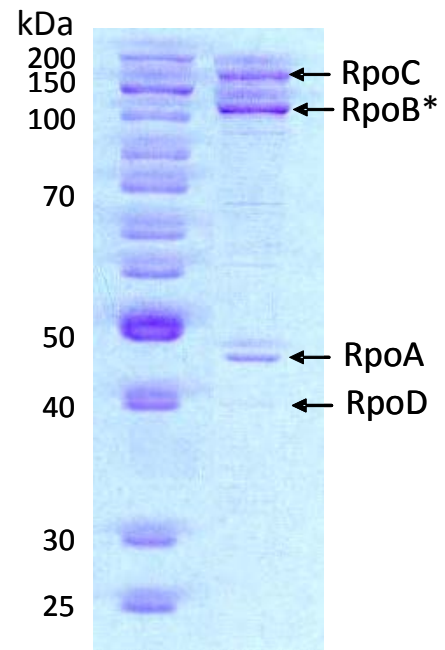

Supplement: Figure S1 — Purification of RpoB partners. SDS-PAGE analysis of protein complexes purified from GY12809 strain. The tagged protein is RpoB and purification was carried out from exponential cells. * This band corresponds to the tagged RpoB fused with the calmodulin binding protein. (PDF) [file pone.0056558.s001.pdf]

[METALLTLDTLAK]

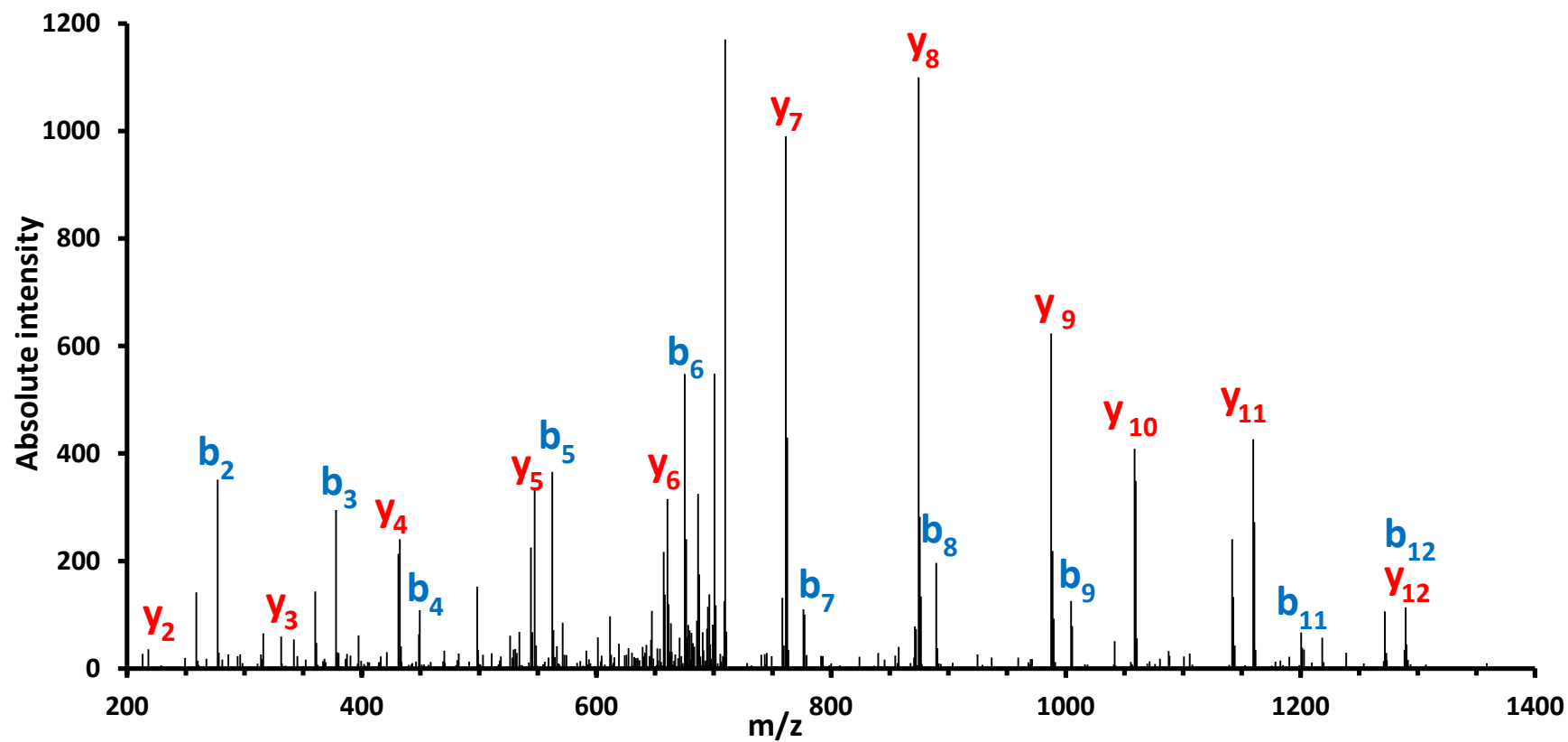

Supplement: Figure S2 — MS/MS spectrum of the peptide [METALLTLDTLAK] corresponding to the N-terminus of DR1245 protein. The mass of the parent di-charged ion was measured at m/z 718.3895 (mass error 0.38 ppm) with an LTQ-Orbitrap XL mass spectrometer (Thermo). The annotated secondary b and y ions are indicated. (PDF) [file pone.0056558.s002.pdf]

*D. radiodurans*

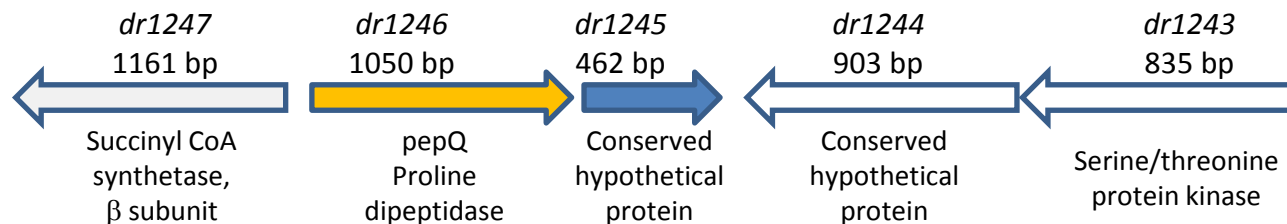

*D. gobiensis*

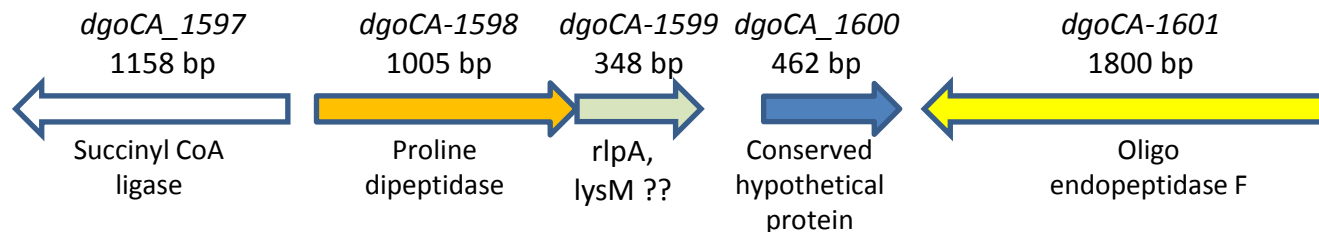

*D. deserti*

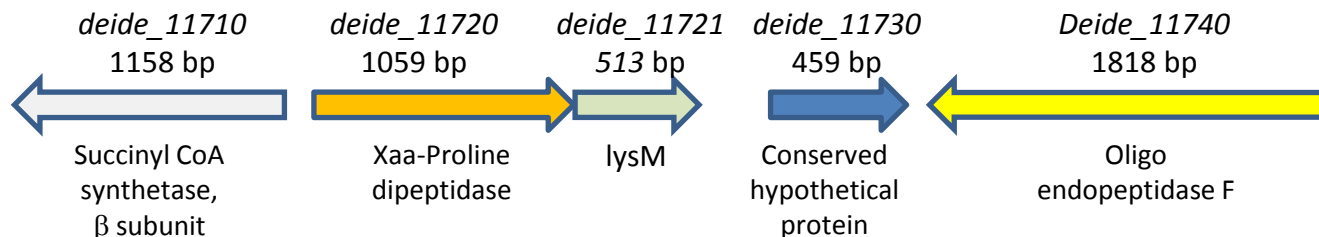

*D. geothermalis*

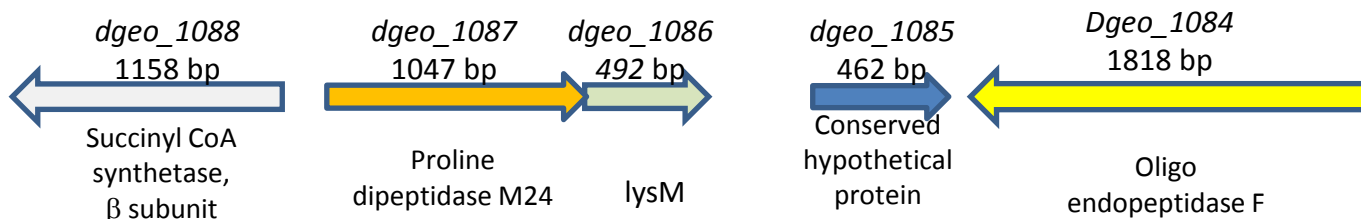

*D. proteolyticus*

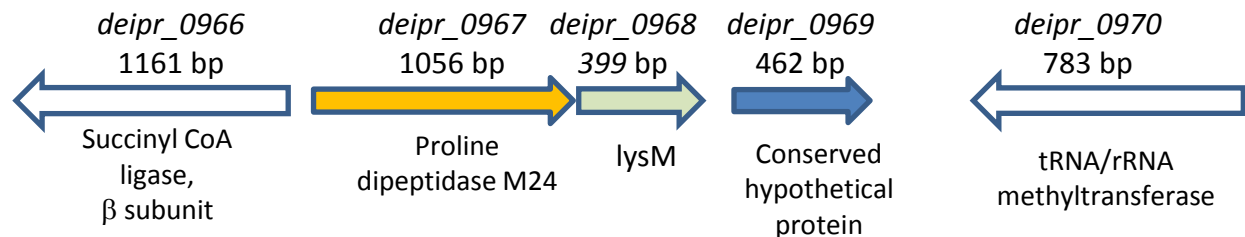

Supplement: Figure S3 — Genome context of deinococcal dr1245 homologous genes. dr1245 homologues from sequenced Deinococcacae (D. radiodurans, dr1245; D. gobiensis, dgo_CA1600, D. deserti, deide_11730; D. geothermalis, dgeo_1085; D. proteolyticus, deipr_0969) were colored in dark blue whereas, the neighbouring proline dipeptidase, lysM, and, oligoendopeptidase F genes were colored in orange, grey and yellow, respectively. (PDF) [file pone.0056558.s003.pdf]
